# Influence of Protective Colloids on Calcium Tartrate Stability and the Astringency Perception in a Red Wine

**DOI:** 10.3390/foods13193065

**Published:** 2024-09-26

**Authors:** Matías Cisterna-Castillo, José Ignacio Covarrubias, Marcela Medel-Marabolí, Alvaro Peña-Neira, Mariona Gil i Cortiella

**Affiliations:** 1 Department of Agro-Industry and Enology, Facultad de Ciencias Agronómicas, Universidad de Chile, Santa Rosa 11315, La Pintana, Santiago 8820000, Chile; matias.cisterna@ug.uchile.cl (M.C.-C.); mmedel@uchile.cl (M.M.-M.); apena@uchile.cl (A.P.-N.); 2 Department of Agricultural Production, Facultad de Ciencias Agronómicas, Universidad de Chile, Santa Rosa 11315, La Pintana, Santiago 8820000, Chile; jcovarru@uchile.cl; 3 Instituto de Ciencias Aplicadas, Facultad de Ingeniería, Universidad Autónoma de Chile, Av. El Llano Subercaseaux 2801, San Miguel, Santiago 8910060, Chile

**Keywords:** carboxymethylcellulose, potassium polyaspartate, Arabic gum, metatartaric acid, mannoprotein, temporal dominance of sensations (TDSs)

## Abstract

Calcium tartrate instability in wines has been a neglected topic for many years. However, it seems that this problem is gaining prominence, and the industry welcomes inputs to address this issue. Among the alternatives that winemakers use for tartrate salt stabilization, the addition of authorized protective colloids is one of the best choices because they are easy to apply and have a low energetic cost. In the present study, the same red wine was treated with five different commercially available protective colloids in triplicate. The effectiveness of such colloids on calcium tartrate potential instability was estimated, in addition to their side effects on the phenolic composition of the treated wines and their astringency perception, as assessed by sensory analyses of the treated wine. The results show that, under these trial conditions, carboxymethylcellulose is the best choice for reducing the risk of calcium tartrate precipitation in wine. Moreover, the application of protective colloids to the wines had little effect on their color, phenolic composition, or evolution during one year of bottle storage. Finally, the addition of protective colloids did not impact the astringency intensity, but it influenced the dynamic perception of astringency according to the temporal dominance of sensation analysis.

## 1. Introduction

Wine is a traditional and popular alcoholic beverage consumed worldwide. For instance, the value of wine exports in 2022 reached 37,600 million (EUR) according to the annual report of the International Organization of Vine and Wine (OIV) [1]. Given the importance of the global wine trade, ensuring the stability of bottled wines is mandatory to avoid consumer rejection and importer claims. Among all the instability sources that can affect wines, the main sources are the precipitation of tartaric acid salts [2,3], such as potassium hydrogen tartrate (KHT) and calcium tartrate (CaT).

Traditionally, the main concern regarding the instability of tartaric acid salts in wines is the precipitation of KHT, which is the most common of these precipitates, which is logical since potassium is the main cation (K^+^) and tartaric acid is the main organic acid in both grapes and wines. In addition, the solubility of KHT decreases when the degree of alcohol increases, and consequently, the wines are supersaturated with KHT, becoming unstable. However, an increase in the occurrence of CaT precipitates in commercial wines has been observed in recent years, which is a concern for winemakers. Moreover, wineries do not have a reference method for evaluating CaT stability in wines, as occurs with KHT stability, which is usually evaluated by using the mini-contact test [4]. Thus, most wineries bottle their wines without being certain about CaT wine stability, increasing the risk of precipitation in the bottle during storage and shipping [5].

To achieve tartrate salt stabilization, winemakers use several tools, such as cold treatment; cold treatment with seeding; ion exchange; electrodialysis; and the use of protective colloids, such as metatartaric acid (MTA), carboxymethylcellulose (CMC), potassium polyaspartate (KPA), mannoproteins (MPs), or Arabic gum (AG) [6,7,8]. Compared with the other mentioned techniques, employing protective colloids to stabilize the tartrate salts in commercial wines is economically favorable since it has a low energy cost and does not require specific equipment [9]. However, the efficacy of these protective colloids has been demonstrated for preventing KHT instability [10,11,12,13], but less information is available about their effectiveness in preventing CaT precipitation [14,15,16,17].

On the other hand, the use of protective hydrocolloids as stabilizers to prevent the precipitation of tartrate salts could influence the sensory perception of treated red wines, given that it has been demonstrated that polymeric substances such as polysaccharides can modulate the astringency perception of red wines [18,19]. Thus, employing protective colloids to achieve tartaric salt stability could have a side effect on the sensory attributes of wines.

Considering all this background, a study was performed by treating the same calcium tartrate-unstable red wine with five different commercial protective colloids in order to investigate its effectiveness on preventing precipitation and, in addition, the side effects of such treatments on the color and phenolic contents of wines. Moreover, the present trial also studied the impact of treating red wines with such protective colloids on the astringency perception. Although the present study was performed by treating a single wine, the results could help the winemakers better understand the extent of applying protective colloids to prevent calcium precipitations. Thus, the present study investigated the influence of employing five different protective colloids (MTA, CMC, KPA, MP, and AG) on CaT stability and their side effects on the perception of astringency in Carménère cv. red wine.

## 2. Materials and Methods

### 2.1. Raw Wine for the Trial

Grapes of the cultivar Carménère from the William Fevre vineyards, located in Pirque (Maipo Valley), were mechanically harvested (April 20th) at technological maturity (22.8 °Brix) during the 2022 harvest season. Approximately 550 kg of harvested grapes were immediately transported to the pilot plant of the Department of Agroindustry and Enology of the University of Chile (La Pintana, Santiago de Chile, Chile), where they were destemmed and crushed (Top 5 Inv., EnoVeneta, Vinicas SA, Chile) and placed in an open food-grade plastic bulk container (660 L). The crushed grapes were immediately inoculated with 0.2 g/kg commercial *Saccharomyces cerevisiae* yeast strain (Lalvin QA23™, Lallemand Inc., Santiago de Chile, Chile) and placed in a thermoregulated room at 20 °C. The alcoholic fermentation was controlled by daily measurements of density and temperature employing a portable density meter model Densito 30PX (Mettler Toledo, Precision, Santiago de Chile, Chile). The cap was gently punched down daily until juice reached 1000 mg/mL density units. The end of alcoholic fermentation was considered when the density remained constant for two consecutive days and the reducing sugar content was less than 2 g/L. At the beginning of the tumultuous phase of alcoholic fermentation, 30 mg/L calcium (in the form of pure CaCO_3_) was added to ensure that the raw wine was enriched in calcium but minimized the impact on the anionic composition of the wine due to its high levels of dissolved CO_2_. After two weeks of maceration, the crushed fermented grapes were pressed by using a vertical press (Pressa 80 SE, EnoVeneta, Vinicas SA, Santiago, Chile), and the wine was racked into a 500 L stainless steel tank inoculated with 0.01 g/L *Oenococcus oeni* bacteria (Lalvin VP41™, Lallemand Inc., Santiago de Chile, Chile) and kept at 22 °C until the end of the process. The advance of the malolactic fermentation was evaluated weekly using paper chromatography [20]. Briefly, the paper chromatography was made using Whatman 1 paper and a solvent prepared by mixing (2:1) a solution of butanol (with 1 g/L of bromophenol blue) with a 50% aqueous solution of glacial acetic acid (Sigma–Aldrich, Santiago de Chile, Chile). Pure malic acid and lactic acid were used as standards (Sigma–Aldrich) and were prepared at 1% (*w*/*v*) employing an hydroalcoholic solution (12% vol) as a solvent. Once the lactic acid bacteria depleted the malic acid in the wine, it was sulfited (0.2 g K_2_S_2_O_5_/L) and placed in a thermoregulated chamber at 4 °C for two weeks to favor KHT precipitation.

### 2.2. Protective Colloid Treatments

The bulk wine was transferred to 18 food-grade polyethylene jugs (10 L) to have 6 conditions in triplicate: control (untreated) wine (C), wine treated with 7.5 g/100 L metatartaric acid (Metavimon, Agrovin, Navarro and CIA, Santiago de Chile, Chile) (MTA), wine treated with 75 mL/100 L potassium polyaspartate (Zenith^®^ One, Enartis, Olivar, Chile) (KPA), wine treated with 75 mL/100 L a solution of carboxymethylcellulose (Estabicel, Agrovin, Navarro and CIA, Chile) (CMC), wine treated with 15 g/100 L yeast mannoproteins (Manolees™, Lallemand, Chile) (MP), and wine treated with 150 mL/100 L Arabic gum (Gomasol Pro, Agrovin, Navarro and CIA, Chile) (AG). Each protective colloid was applied at the average dose recommended by the manufacturer, and the legacy thresholds were considered. The wines were kept in plastic jugs for 10 days, after which the wines were racked and bottled in 750 mL green glass bottles, capped with natural corks (Cristal^®^ V, Bourrassé Chile SA, Santiago de Chile, Chile), and stored horizontally in a dark undergrown cellar (16–18 °C) until analysis.

### 2.3. General and Polyphenolic Analyses of the Wines

The alcohol content, titratable acidity, and pH were measured following the methods recommended by the OIV [21]. Moreover, color and phenolic composition of wines were estimated to assess the effect of protective colloids on the evolution of phenolic contents during wine aging, and to check possible relationships between phenolic composition and the sensory perception of astringency. The color of the wines was assessed by measuring the absorbance at wavelengths of 420 nm, 520 nm, and 620 nm with a quartz cuvette (1 mm pathlength). The color intensities were calculated as the sum of the absorbances at 420 nm, 520 nm, and 620 nm for each wine, and the resulting number was multiplied by 10 to refer to the result for a 10 mm pathlength cuvette. Hue values were computed as the quotient between the absorbance at 520 nm and the absorbance at 420 nm [22]. Total tannins were estimated by using the methyl cellulose precipitation method [23]. Total anthocyanins were estimated by the anthocyanin assay, and total phenols were estimated by measuring the OD 280 value [22] and using a calibration curve with gallic acid as an external standard.

### 2.4. Tests for Determining Tartrate Salt Stability in Wines

To assess the potential stability of wines regarding tartaric salts, the following analytical tests were applied to determine the KHT and CaT stabilities one month after bottling. Moreover, a bottle of each wine was stored in an underground cellar (16–18 °C) for one year, filtered with a polymeric S-Pak membrane (mixed cellulose esters, pore size 0.45 µm, diameter 47 mm, Merck–Millipore, Santiago de Chile, Chile), and washed with cold absolute ethanol (0 °C) to identify the occurrence of crystals.

#### 2.4.1. Mini-Contact Test

To assess the potential stability of KHT, the mini-contact test (Martin Vialatte Company variant) was applied as reported by [4], considering a 3% conductivity loss as the stability threshold [9]. The micronized potassium bitartrate was purchased from Sigma–Aldrich, and the conductivity measurements were performed with a HANNA HI 5321 conductometer (HANNA Instruments Chile, Santiago de Chile, Chile).

#### 2.4.2. Abguéguen and Boulton Test

To assess the potential stability of CaT, the test proposed by Abguéguen and Boulton was applied [24], employing the Enocristal Ca (Enartis) as a pure, commercially micronized CaT crystal as a seed for crystal growth. Briefly, 10 mL of the initial sample was stored in a 50 mL Pyrex bottle and saved (as initial wine) for subsequent analysis of the initial Ca concentration [Ca]_0_. Moreover, 10 g/L CaT micronized crystals were applied to a sample of 100 mL cold wine (2 °C) and maintained with magnetic stirring for two hours. Subsequently, the sample was filtered with a syringe-driven filter unit (0.45 µm). When the samples recovered at room temperature, 10 mL of the filtered wine was placed in a Pyrex bottle of 50 mL (to determine the final Ca concentration [Ca]_f_). The calcium contents of both samples (initial and final wines) were analyzed using MP-AES 4200 microwave plasma atomic emission spectroscopy (Agilent Technologies, Arquimed S.A., Santiago de Chile, Chile) after digestion (mixing the wine with 30% H_2_O_2_ and 65% HNO_3_ (5:1:3) and subjecting the mixtures to an autoclave cycle (LabTech, Daihan Labtech Co., Ltd., Namyangju-City, Republic of Korea) as previously reported [25]. The variation in Ca during the test was expressed as a percentage relative to the initial concentration, and the stability threshold was established at a 5% Ca decrease, as suggested by Abguéguen and Boulton [24].

### 2.5. Soluble Polysaccharide Contents and Protective Colloid GPC Profiles

#### 2.5.1. Wine-Soluble Polysaccharide Profiles

Wine-soluble polysaccharide levels were estimated as previously reported by employing the HRSEC-RID method after precipitation with cold acidified ethanol [26]. Briefly, 10 mL of wine was concentrated to 2 mL using a Labconco CentriVap concentrator (Merck). Ten milliliters of cold acidified ethanol (0.3 M HCl) were added to the concentrated wine, and the sample was stored at 4 °C for 24 h. Subsequently, the sample was centrifuged (at 8500 rpm for 10 min, 4 °C), and the supernatant was discarded. The pellets were washed with cold absolute ethanol twice, redissolved in ultrapure water (1 mL), placed in Eppendorf tubes, and stored at −80 °C. Finally, the samples were freeze-dried using a FreeZone Legacy 2.5 L freeze dryer (Labconco, Merk-Millipore, Santiago de Chile, Chile), and the obtained lyophilizates were dissolved in 1 mL of aqueous ammonium formate (30 mM), filtered with syringe-driven filter units (0.45 µm pore size, Millex^®^–GV, Merck Millipore Ltd., Santiago de Chile, Chile), and injected into an Agilent 1260 Series chromatograph equipped with an autosampler, a quaternary pump, a column oven, and a refractive index detector (Agilent Technologies). The chromatographic conditions included isocratic flow (0.6 mL/min) using aqueous ammonium formate (30 mM) as the mobile phase and two columns connected in series (OHpak SB-803 HQ and OHpak SB-804 HQ, Shodex, Showa Denko, Sumilab LTDA, Santiago de Chile, Chile) as the stationary phase. The column oven was kept at 20 °C, and the injection volume was 100 µL. The columns were calibrated with dextran standards from *Leuconostoc mesenteroides* of different molecular masses (Sigma–Aldrich), and the calibration was performed by employing the average number molecular mass (Mn) expressed in kDa. The estimation of polysaccharide concentrations was performed employing dextran (410,000 from *Leuconostoc mesenteroides*, Sigma–Aldrich) and pectin (esterified potassium salt from citrus fruit, 20–34% esterified, Sigma–Aldrich) as external standards.

#### 2.5.2. GPC Profiles of the Protective Colloids

The same chromatographic conditions employed for soluble polysaccharide analysis were also employed for the characterization of the molecular size distribution of the employed commercial protective colloids. To establish the direct GPC profile of the protective colloids, each colloid was dissolved in 30 mM ammonium formate at a concentration of 1 g/L and injected after filtration (0.45 µm). To establish the GPC profile of the precipitable protective colloids, each sample was dissolved in a wine-like solution (12.5 vol%, 5 g/L tartaric acid, pH = 3.5) in triplicate at the same concentrations applied to the wines during the treatments. After that, the samples were precipitated using cold acidified ethanol in the same way as the wines.

### 2.6. Sensory Analysis

Fourteen panelists (6 women and 8 men) analyzed the wine samples. The panelists signed an informed consent about their participation in the study and joined voluntarily the panel (without any financial compensation), knowing that they could withdraw at any time. The selected tasters were of legal drinking age, regularly consumed wine, and had experience tasting wines. The data from each panelist were blind-treated without keeping the individual results from each subject. The panel accounts with winemakers, academics, and students of the Agronomical Sciences Faculty of the University of Chile, all of whom are regular wine consumers. The panelists were trained for astringency perception as previously described [27], including the use of different hand tactile textures associated with the mouth-feel selected adjectives (Section 2.6.2). In order to minimize potential bias, all the wine samples were identified by a numeric code of three aleatory digits and were tasted at 16–18 °C, employing ISO official tasting glasses poured with 30 mL of wine. After the training, a ranking test was performed to validate the panel, ordering the astringency intensity of five samples that consisted of the same raw wine employed for the protective colloid trial with increasing additions of oenological tannins (0, 0.1, 0.5, 1.0, and 2.0 g/L). The five samples were identified by using a numeric code of three random digits, and every panelist tasted the samples in a different order. The panel obtained a H_a_ value of 736, which is greater than the reference H_0_ value of 661 (for 14 panelists and 5 samples); thus, the panel was considered able to discriminate among samples with different intensities of astringency [27,28].

#### 2.6.1. Intensity of Astringency by Descriptive Analysis

The attribute “astringency”, considering the intensity of the perception, was scored by the panelists using a 15 cm unstructured line (including a label that indicates the intensity and direction above each end of the line).

#### 2.6.2. Astringency Temporal Profiles

Wine astringency was also assessed by temporal dominance of sensations (TDS) analysis following a reported method [27] to assess the sub-attributes of astringency perception. This analysis focuses on the dynamic perception of astringency, considering the following adjectives, namely, soft, mouth coat, adhesive, drying, and aggressive, to describe astringency perception according to the definitions established in the mouth-feel wheel [29]. The TDS analysis was performed using FIZZ software V 2.1 (Biosystemes, Couternon, France), and the tasters were taught employing this methodology. The tasting sequence and protocol used for the sensory evaluation of the samples are shown in Table 1.

The dynamic profiles were recorded by using FIZZ software, and in addition to the profiles, for each sample, the maximum dominance rate (%) and the time (s) at which this maximum dominance rate was reached for each attribute and sample were determined. Moreover, the period (s) during which an attribute was dominant (when it overtakes the chance level line) was also calculated for each attribute and wine sample [30].

### 2.7. Statistical Analysis

All the chemical and physical results are presented as the average ± standard deviation of triplicate analyses for each condition, as well as the astringency intensity rates. Moreover, a one-way analysis of variance (*p* < 0.05) followed by a post hoc test (Tukey) was conducted for multiple comparisons employing GraphPad Prism v 10.2.1 (GraphPad Software LLC, Boston, MA, USA). The TDS data were collected and tabulated with FIZZ software, and the charts of the temporal profiles of astringency were depicted employing the same version of GraphPad Prism mentioned before.

## 3. Results and Discussion

### 3.1. Effect of Protective Colloids on the Color and Chemical Composition of Treated Wines

Although the main reason for using protective colloids is to achieve tartaric salt stability, it is relevant to determine the effects of such additives on the features of the resulting wines.

#### 3.1.1. Impact of Protective Colloids on the General Parameters of Wine

It is well known that pH deeply influences the CaT potential stability of the wines, due to the larger proportion of tartrate ions (T^−2^) at higher pH values [14]. Hence, a variety like Carménère is suitable for studying the CaT instability due to its usual low acidity and high pH, given that it used to be harvested late in the season to prevent an excess of pyrazine character [31]. The wine employed for this trial had a pH near 4.0 (Table 2), which enhances the potential CaT instability. As shown in Table 2, protective colloid application to the wines had some impact on the titratable acidity and pH, while no effect was observed on the alcoholic degree of treated wines. Wines treated with MTA had the lowest pH and the highest titratable acidity. These results could be related to partial hydrolysis of the metatartaric acid and the resulting release of tartaric acid molecules [4]. In contrast, the MP- and AG-treated wines had lower titratable acidities than did the untreated (C) wines. The impact of application of protective colloids on wine pH can also indirectly affect its effectiveness, given that the pH can affect the charge density of the colloids, and it has been described that higher charge density leads to best ability to complex calcium ions and, accordingly, its effectiveness preventing CaT precipitation [14]. 

#### 3.1.2. Influence of Protective Colloids on the Color and Phenolic Composition of Wines

**Figure 1:**
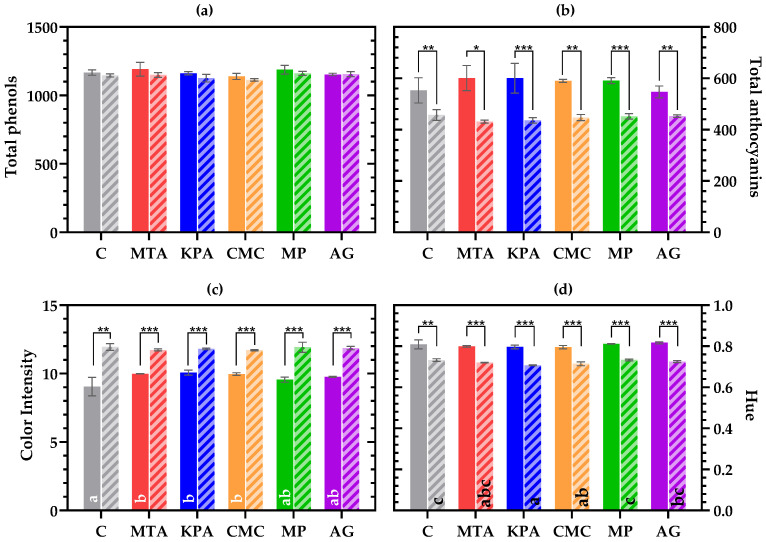
The solid bars correspond to the analyses 1 month after bottling. Striped bars correspond to the analyses 1 year after bottling. Different letters in the bars indicate significant differences (*p* < 0.05) among treatments for the same sampling time. Differences between sampling times are indicated as follows: (*): *p* < 0.05; (**): *p* < 0.01; and (***): *p* < 0.001. (**a**) Total phenols expressed in mg/L gallic acid equivalents. (**b**) Total anthocyanins expressed in mg/L malvidine-3-*O*-glucoside equivalents. (**c**) Color intensity expressed in absorbance units relative to a 10 mm path length cuvette. (**d**) hue of the wines (Abs_420nm_/Abs_520nm_).

It has been reported that, in addition to their phenolic content, protective colloids can affect color stability in wines. To assess the influence of the employed colloids, the wine color, anthocyanin content, and total phenolic content were analyzed one month and one year after bottling, and the results are shown in Figure 1.

The total phenolic content was not affected by the protective colloid used or by the storage time. This result seems to indicate that the commercial colloids employed in this trial have a low effect on the total phenolic content of wine and its stability over a relatively intermediate duration since all the treated wines showed the same concentration as the control untreated wine. No differences in the anthocyanin content were observed depending on the treatment. However, the total anthocyanins decreased during the storage time for all the treated wines to the same extent. These results seem to indicate that the protective colloids employed in this trial had a low impact on the anthocyanins either early after treatment or during bottle storage. The low impact of using protective colloids such as CMC, KPA, or MP on the phenolic compound composition has been previously reported [11,32,33]. However, it should be noted that the influence of protective colloids also depends on the wine matrix, and, hereby, it is possible that employing the same treatments in different wines leads to different results. 

Early after the treatment, the control wines showed a significantly lower color intensity (CI) than the wines treated with MTA, KPA, and CMC. However, such differences disappeared after one year of bottle storage due to an increase in the CI for all the wines. The increase in CI during early aging has been related to the formation of polymeric pigments (through the reaction between anthocyanins and proanthocyanidins) and anthocyanin derivatives, which cause a hyperchromic shift that enhances the color intensity of red wines [34]. The same reactions of free anthocyanins also explain the evolution of hue (corresponding to the relation between the yellow and the red components of the wine) during storage, given that the formation of anthocyanin derivatives can also induce a bathochromic shift that could explain the decrease in hue values for all the wines throughout the aging time. Hue did not show significant differences among treatments after 1 month of storage, but after 1 year, the control and MP wines showed the highest hue values, while KPA wines showed the lowest. Thus, it seems that the effect of the employed protective colloids on the evolution of wine color during the first year of wine storage is very limited, as reported previously by other authors [11,32,33].

### 3.2. Effect of Protective Colloids on the Tartaric Salt Stability in Wines

**Figure 2:**
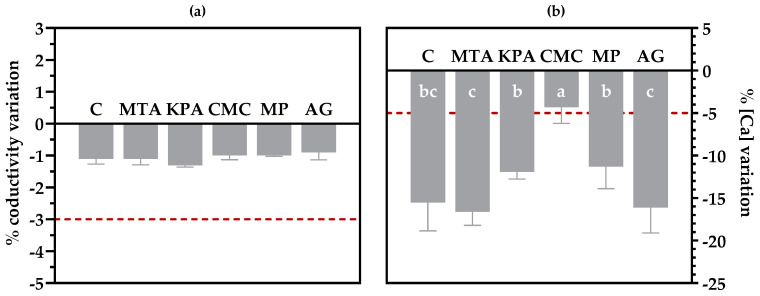
Results of the stability tests of the wines. (**a**) Conductivity variation during the mini-contact test. (**b**) Calcium concentration variation during the Abguéguen and Boulton test. The dashed line indicates the stability threshold for each test. The different letters above the bars indicate significant differences (*p* < 0.05) among the treatments.

As mentioned in Section 2.1, the treated and untreated (C) wines were subjected to cold treatment to stabilize the wines, and such treatment was effective given that all the wines were stable for KHT precipitation according to the mini-contact test, as shown in Figure 2a. As wines are stable considering the KHT salt, any natural crystalline precipitation occurring in the wines during storage should be related to CaT precipitation. Moreover, the effectiveness of protective colloids against KHT precipitation was amply demonstrated [6,12,32,35,36], and the commercial wines that presented problems related to Ca precipitation were KHT stable before bottling. Thus, the present experimental design focuses on the occurrence of CaT precipitates, excluding the well-known effect of protective colloids on KHT crystallization.

Considering the CaT potential stability of the wines, Figure 2b shows that only the wines treated with CMC were stable according to the Abguéguen and Boulton test, which demonstrates the inefficacy of cold treatment to allow CaT stability in wines, as has been widely accepted [8]. These results seem to indicate that, among the tested protective colloids under these experimental conditions, CMC is the only one with an actual protective effect against CaT crystallization in wines. This protective effect of CMC is probably related to its capacity to chelate Ca^2+^ cations [37,38], as has also been previously reported for polysaccharides made with uronic acids [14,15,38], or recently described for the sulphated hydrocolloid Carrageenan, able to stabilize the CaT of white and rosé wines [16]. In addition, after one year of storage, the CMC-treated wines did not show evidence of crystalline precipitates after membrane filtration, in contrast to what occurred with the other samples to a greater or lesser extent, which confirms that the Abguéguen and Boulton test could be a good tool to predict natural CaT instability in wines.

Moreover, the Ca decrease in protective colloids other than CMC did not significantly differ from that in untreated (C) wines during the test, indicating that, under the employed conditions, MTA, KPA, MP, and AG did not stabilize the wines, but neither increased the CaT instability as reported by other authors [14,17]. Therefore, it seems that the wine matrix deeply influences the effectiveness of protective colloids considering CaT precipitation, especially in the case of red wines [14]. Thus, our results seem to point out that CMC could be a good solution to stabilize red wines with high pH, deep color, and great phenolic contents. 

### 3.3. GPC Profiles of the Employed Protective Colloids

**Figure 3:**
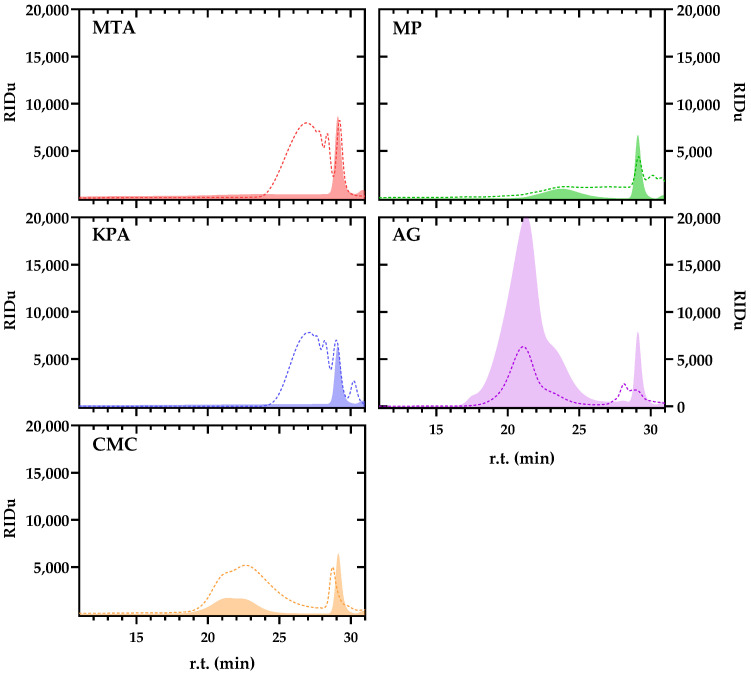
GPC profiles of the protective colloids employed during the trial. Dashed profiles correspond to the direct injection of each protective colloid into the HRSEC-RID system after dissolution with the mobile phase (1 g/L). Shady profiles correspond to the precipitable profile of each protective colloid dissolved in wine-like solution at the same concentration applied during treatment and analyzed like wines.

It has been reported that the characteristics of protective colloids can affect their effectiveness [39]. For that reason, the Gel Permeation Chromatography (GPC) profiles of each employed protective colloid were characterized, and the profiles are shown in Figure 3.

The direct GPC profiles (dashed lines, Figure 3) of MTA and KPA are quite similar, corresponding mainly to a continuous and highly polydisperse fraction (Mn ranging from approximately 36 kDa to 1.5 kDa) overlapped with three less polydisperse fractions (approximately 4.0 kDa, 3.0 kDa, and 1.5 kDa, respectively). In contrast, two fractions (made up of the overlap of different fractions since they do not show a Gaussian distribution) can be distinguished for CMC (the first ranging from 600 kDa to 4 kDa and the second from 4 kDa to 1.5 kDa) and AG (the first ranging from 1800 kDa to 12 kDa and the second from 7 kDa to 1 kDa). Finally, the GPC profiles of the MPs corresponded to several fractions that overlapped each other, ranging from 340 kDa to 1 kDa.

It has been described that the chemical features of the protective colloids influence their efficacy to inhibit the KHT crystallization. For instance, some published data point out that the effectiveness of CMC is lower when its mean degree of polymerization increases [32,40], given that larger chains could favor folding and reduce its interaction with sub-critical crystal nuclei, avoiding KHT crystallization. In contrast, other studies point out that CMC efficacy in reducing KHT instability is more related to CMC viscosity than with the CMC polymerization degree itself [39]. The CMC employed in the present trial showed two fractions with an averaged M_n_ of 147 KDa and 58 KDa, respectively; those are in the range of molecular sizes reported as effective CMCs in synthetic solution and red wines to reduce the potential crystallization of KHT [32,39,40]. However, such studies focused on the KHT crystallization, and the wine employed in the present study was stable from the KHT point of view. Therefore, more research is required to assess the influence of the structural characteristics of protective hydrocolloids on their efficacy in preventing CaT precipitation in bottled wines.

### 3.4. Precipitable Colloid Analysis of Wines

**Figure 4:**
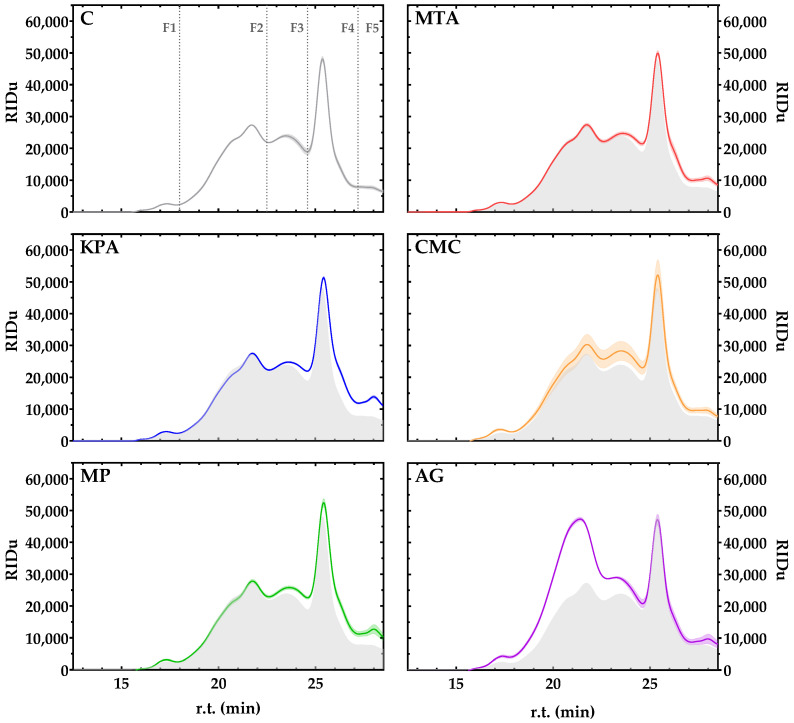
Averaged GPC profiles of ethanol precipitable colloids of wines for each treatment. The untreated wine chromatogram (C) shows the fraction ranges employed for quantification (Table 2), and its profile is maintained (gray shadow) throughout the charts of the treated wines.

The soluble polysaccharides of wines have been frequently estimated by HRSEC-RID after precipitation with cold acidified ethanol since few wine proteins precipitate under these conditions. The characterization of the polysaccharide fractions of the treated wines according to this methodology is shown in Figure 4 and Table 3.

Wines treated with AG had the greatest concentration of the larger polysaccharides (F1 and F2, with molecular masses greater than 100 kDa), especially in the case of F2, which had the greatest concentration and the largest M.W. range. For the medium molecular mass fraction (F3, approximately 50 kDa), the CMC wines had the highest concentration. In contrast, KPA wines had the highest concentration for low molecular mass fractions (F4 and F5, lower than 25 kDa). In general, C (untreated wine) had the lowest polysaccharide concentration among all the fractions, as can also be observed visually in the chromatographic profiles shown in Figure 4. However, in this case, the increase is not related to a modification of the naturally occurring polysaccharides of the wine (mainly pectic substances from grapes and mannoproteins from yeasts) but rather to the incorporation of exogenous protective colloids into the wine matrix. To prove this, each protective colloid was added to a wine-like solution in the same way as in the treated wine, and the profiles after cold ethanol precipitation (shaded profiles, Figure 3) match perfectly with the described increase in treated wines with respect to the untreated one.

**Figure 5:**
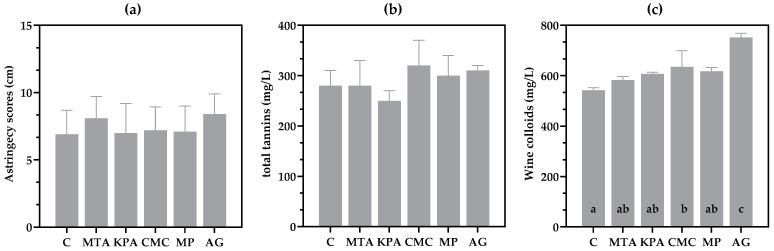
(**a**) Astringency scores obtained for each wine through an unstructured scale of 15 cm. (**b**) Tannin content of wines expressed as mg/L catechin equivalents. (**c**) Wine-soluble colloid contents expressed as mg/L polysaccharide equivalents. The different letters above the bars indicate significant differences (*p* < 0.05) among the treatments.

As a result, the total colloidal contents of the wines differed significantly, with the highest for those treated with AG and the lowest for the untreated (C) wines (Figure 5c).

### 3.5. Astringency Sensory Evaluation

As mentioned previously (Section 3.1), to justify the chemical characterization of treated wines, regardless of the reason for applying the protective colloids (tartaric salt stabilization), if applied, they were incorporated into the wine matrix and therefore could impact the sensory perception of the wines.

One of the main sensory attributes of red wine is astringency, given that its perception can affect consumer acceptability. The perception of astringency is largely related to the phenolic composition of wines, playing a special role in the tannins [41]. However, the phenolic composition does not always explain the sensory differences among wines [42]. Moreover, it has been reported that hydrocolloids such as native wine polysaccharides influence the sensory perception of astringency [41]. Thus, it could be hypothesized that employing protective colloids can affect the astringency perception of wines.

#### 3.5.1. Astringency Intensity

When the panelists rated the astringency intensity of the wine samples on an unstructured scale, the scores obtained were statistically the same, as shown in Figure 5a. This lack of differences in the astringency scores agrees with the lack of significant differences in the tannin contents of the wines (Figure 5b). However, wine astringency is a complex phenomenon [41], and its perception involves several mouth-feel sensations that are not necessarily static; hence, reducing the perception of astringency to a single score may oversimplify the phenomenon. This was the main reason for performing a dynamic sensory analysis of astringency perception.

#### 3.5.2. Astringency TDS

**Figure 6:**
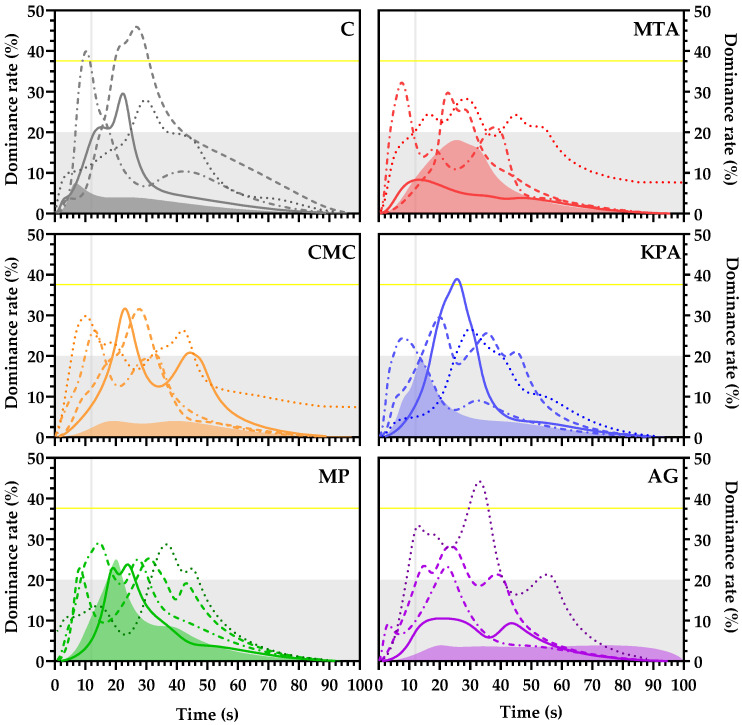
TDS profiles of the treated wines. TDS profiles of the treated wines according to the adjectives: (dotted line): soft; (dash–dotted line): mouth coating; (dashed line): adhesive; (solid line): drying; (shaded profile): aggressive. The gray shaded (horizontal) part of the graph (under 20% of dominance) corresponds to the zone below the chance level. The yellow horizontal line corresponds to the significance level. The vertical gray line (12 s) corresponds to the time at which the samples were spit out.

To obtain a dynamic description of astringency perception, Temporal Dominance of Sensations (TDS) analysis was performed since it has been previously reported that wines with similar tannin contents and with the same intensity rates according to descriptive analysis can have different dynamic profiles [43]. For that reason, the TDS profiles (Figure 6) of treated wines were built considering the dominance rate of each of the five attributes (soft, mouth coating, adhesive, drying, and aggressive) employed for astringency perception characterization.

Indeed, the dynamic profiles of wine astringency considering the selected sub-attributes are different for wines treated with different protective colloids. As shown in Figure 6, the perceptions of astringency on the mouth coating and adhesive surfaces reached dominance rates above the significance level for the C samples. In contrast, only the drying perception reached a significant dominance rate for the KPA samples, and the soft attribute reached a significant dominance rate for the AG samples. For the other treatments (MTA, KPA, CMC, and MP), none of the sub-attributes surpassed the significance level. Given that the phenolic composition of the wines is almost the same, the differences in the dynamic profile of the astringency could be related to the soluble colloidal contents of the wines.

**Figure 7:**
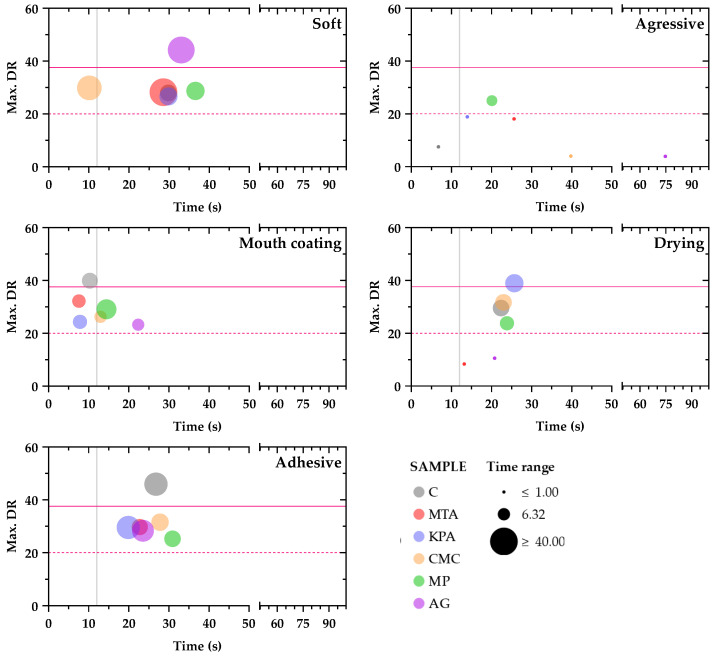
Maximum dominance rate (%, Y-axis) and occurrence time (s, X-axis) for each attribute and sample obtained through TDS analysis. The bubble sizes are proportional to the time frame in which the attribute is dominant (surpassing the chance level). The dashed horizontal line corresponds to the zone in which the attributes are not dominant (below the chance level). The solid horizontal line corresponds to the significance level. The vertical solid line indicates the time the wine sample was discarded according to the TDS protocol employed.

Moreover, Figure 7 contains information about the maximum dominance rate for each attribute (Max. DR (%)) and its reaching time (s) per sample. Furthermore, the bubble size is proportional to the period in which each attribute remains dominant (it surpasses the chance level). 

According to these data, the least frequent attribute selected by panelists was “aggressive”, which was dominant only for MP wines approximately 20 s after the beginning of the analysis. The “drying” attribute was dominant for the C, CMC, MP, and KPA wines, and its maximum dominance rate was between 20 and 25 s, being significantly dominant only for the KPA wines. The “mouth coating” attribute reaches its maximum dominance rate earliest, when panelists still had the wines in the mouth for the C, MTA, and KPA wines, and just after discarding the samples for the CMC and MP wines. In contrast, it seems that the addition of AG delays the maximum dominance rate for “mouth coating”, since it was reached after 20 s of analysis. Moreover, only the C wines reached a significant dominance rate for this attribute (approximately 10 s). The “adhesive” attribute reached its maximum dominance rate between 18 and 30 s for all the samples, and it remained dominant for longer periods than did “mouth coating”, “drying”, and “aggressive”. However, only the C wines showed a significant dominance rate for the adhesive attribute, reaching its maximum level at approximately 25 s. Finally, for the “soft” attribute, the maximum dominance rates were reached between 28 and 36 s for all the samples except for the CMC wine, which reached its maximum dominance rate earliest, at 10 s, just before spitting out the sample. Moreover, only the wines treated with AG reached statistically significant dominance rates for this attribute, being dominant through the largest time frame when compared with the other samples and attributes.

Therefore, considering the dynamic perception of wine astringency, the differential colloidal contents of wines could influence the TDS profiles of the samples, regardless of the lack of differences in phenolic composition. Specifically, it seems that adding MTA, CMC, and MP smoothed the “mouth coating” and “adhesive” perceptions of astringency, given that both attributes did not reach the significance level for MTA, CMC, and MP wines while they exceeded the significance level for untreated (C) wines, appearing (more or less) at the same frame time for all the samples. Wines treated with KPA showed a significant maximum dominance rate for the drying attribute at approximately 25 s. Thus, it seems that KPA could enhance the astringency perception of wines since tasters employed a harder adjective to describe the astringency perception (they felt lack of lubrication in the mouth) of this condition when compared with the other ones. Finally, the addition of AG to the wines modified the TDS profile, smoothing the “mouth coating” and “adhesive” perceptions while increasing the “soft” perception of astringency when compared with the untreated (C) wine, which is consistent with reported results about the ability of Arabic gum to reduce the astringency perception [19]. Furthermore, the increase in the “soft” perception of astringency could be related to the reported ability of Arabic gum to increase the in-mouth attributes of body and weight [44]. Indeed, many winemakers employ the addition of AG to increase the weight in the mouth of some flat wines, regardless of the stabilization requirements. Considering the TDS profile of the AG wines, such applications are logical, given that treating wines with AG increased the “soft” perception dominance rate and its time frame, promoting the feeling of pleasant astringency.

## 4. Conclusions

In view of the obtained results, several conclusions about the use of protective colloids for red wines can be drawn. First, it seems that among the commercially available protective colloids employed during this trial, only CMC exerted a protective effect against CaT precipitation according to the Abguéguen and Boulton test and considering the absence of crystalline material (as seen with the naked eye after filtration through a membrane) after one year of storage. Second, despite the initial reticence to employ colloids such as CMC of KPA to treat red wines after their approval, it seems that the current commercially available preparations have a low impact on the phenolic composition and color features. For instance, after one year of bottle aging, small differences in phenolic composition, color, and evolution trends were observed between untreated wine and wine enriched with protective colloids. Finally, the presence of exogenous protective colloids in the wine matrix did not impact the overall astringency rating of the wines, but it altered their dynamic perception compared to that of the untreated wine according to their TDS profiles.

## Figures and Tables

**Table 1:** TDS of the astringency perception protocol.

Step	Time (s)	Instructions
1	-	Hold the glass with the left hand.
2	0	With the right hand, click in the “Start” button and simultaneously bring all the contents of the glass to the mouth.
3	From 0 to 12	Click on the button that matches the most dominant attribute now among those listed below. Click on a new attribute when you feel a change in the dominant attribute.
4	12	Spit out the wine.
5	From 13 to 30	Continue the evaluation of the dominant attribute.
6	30	Click on the “Stop” button when you do not perceive astringency.
7	100	End of the evaluation.

**Table 2:** Titratable acidity, pH, and alcohol content of the wines. Different letters in the same column indicate significant differences (*p* < 0.05) among treatments.

Treatment	pH	Titratable Acidity ^1^	%Vol.
C	3.98 ± 0.01 ^b^	4.22 ± 0.04 ^bc^	12.2 ± 0.00 ^a^
MTA	3.93 ± 0.01 ^a^	4.25 ± 0.04 ^c^	12.2 ± 0.12 ^a^
KPA	3.93 ± 0.03 ^ab^	4.07 ± 0.04 ^ab^	12.2 ± 0.06 ^a^
CMC	3.98 ± 0.01 ^b^	4.20 ± 0.08 ^bc^	12.4 ± 0.06 ^a^
MP	3.99 ± 0.01 ^b^	4.02 ± 0.09 ^a^	12.2 ± 0.17 ^a^
AG	3.99 ± 0.01 ^b^	4.02 ± 0.05 ^a^	12.4 ± 0.06 ^a^

^1^ Results expressed as g/L of tartaric acid equivalents.

**Table 3:** Characterization of precipitable colloid fractions: concentration (Conc. expressed as mg/L), average molecular mass (Mn, expressed in KDa), and molecular mass range for each fraction (range, expressed in KDa). Different letters in a row indicate significant differences (*p* < 0.05) among treatments.

Fraction		Control	MTA	KPA	CMC	MP	AG
F1	Conc.	8.2 ± 0.4 ^a^	9.9 ± 0.1 ^a^	9.4 ± 0.5 ^a^	12.8 ± 1.6 ^bc^	10.3 ± 1.0 ^ab^	13.1 ± 1.3 ^c^
	Mn	1474 ± 23 ^ab^	1491 ± 12 ^bc^	1506 ± 14 ^bc^	1532 ± 26 ^c^	1506 ± 6 ^bc^	1439 ± 15 ^a^
	Range	3897–1136 ^a^	4216–1111 ^a^	4151–1104 ^a^	4275–1110 ^a^	3936–1101 ^a^	4192–1176 ^a^
F2	Conc.	218.0 ± 3.3 ^a^	215.4 ± 3.4 ^a^	211.2 ± 0.9 ^a^	241.9 ± 27.7 ^a^	217.6 ± 6.1 ^a^	396.6 ± 7.1 ^b^
	Mn	129.0 ± 0.5 ^b^	127.3 ± 0.4 ^ab^	126.2 ± 0.2 ^a^	126.4 ± 0.7 ^a^	126.5 ± 0.4 ^ab^	154.7 ± 2.1 ^c^
	Range	1136–78 ^b^	1111–78 ^a^	1104–78 ^a^	1110–80 ^a^	1101–79 ^a^	1176–70 ^c^
F3	Conc.	124.1 ± 4.6 ^a^	131.6 ± 2.8 ^a^	131.8 ± 1.2 ^a^	153.4 ± 17.1 ^b^	138.9 ± 3.7 ^ab^	135.7 ± 5.5 ^ab^
	Mn	48.3 ± 1.7 ^c^	45.1 ± 0.3 ^abc^	44.6 ± 0.1 ^ab^	47.4 ± 0.8 ^bc^	44.1 ± 0.1 ^a^	54.7 ± 2.2 ^d^
	Range	77.5–25.9 ^b^	78.3–26.0 ^bc^	77.9–26.0 ^b^	79.5–25.8 ^c^	78.8–26.0 ^bc^	69.6–25.4 ^a^
F4	Conc.	165.7 ± 5.5 ^a^	188.7 ± 6.5 ^abc^	206.9 ± 3.8 ^c^	192.2 ± 16.9 ^bc^	206.7 ± 6.8 ^c^	170.7 ± 10.6 ^ab^
	Mn	16.8 ± 0.0 ^d^	16.5 ± 0.1 ^bc^	16.3 ± 0.0 ^a^	16.5 ± 0.1 ^bc^	16.4 ± 0.1 ^ab^	16.7 ± 0.1 ^cd^
	Range	25.9–6.1 ^bc^	26.0–6.1 ^bc^	26.0–5.9 ^c^	25.8–6.0 ^b^	26.0–5.9 ^c^	25.4–6.2 ^a^
F5	Conc.	26.4 ± 2.1 ^a^	37.3 ± 2.1 ^bc^	47.5 ± 1.9 ^c^	34.7 ± 3.8 ^ab^	44 ± 4.7 ^bc^	35.1 ± 6 ^ab^
	Mn	5.6 ± 0.1 ^b^	4.0 ± 0.0 ^a^	3.8 ± 0.0 ^a^	5.0 ± 0.8 ^b^	3.9 ± 0.1 ^a^	3.9 ± 0.1 ^a^
	Range	6.1–3.0 ^a^	6.1–2.9 ^ab^	5.9–2.8 ^a^	6.0–2.9 ^a^	5.9–2.8 ^a^	6.2–2.9 ^b^

## Data Availability

The original contributions presented in the study are included in the article. Further inquiries can be directed to the corresponding author.
